# *Toxoplasma gondii* Infection Causes an Atypical Abundance of Oxytocin and Its Receptor in the Female Rat Brain

**DOI:** 10.3390/pathogens10111495

**Published:** 2021-11-17

**Authors:** Samira Abdulai-Saiku, Ajai Vyas

**Affiliations:** 1 School of Biological Sciences, Nanyang Technological University, Singapore 637551, Singapore; avyas@ntu.edu.sg; 2 Department of Neurology, University of California San Francisco, San Francisco, CA 94110, USA

**Keywords:** Apicomplexan parasites, behavioral manipulation, sex, hypothalamus, nonapeptides, medial amygdala

## Abstract

Infection with the protozoan *Toxoplasma gondii* causes loss of innate fear of cat odors in both male and female rats. This behavioral change is presumed to reflect a parasitic manipulation that increases transmission of the parasite from its intermediate to definitive host. The host behavioral change in male rats is dependent on gonadal steroids. In contrast, the loss of fear in female rats is not accompanied by greater gonadal steroids and cannot be rescued by gonadectomy. This disparity suggests that proximate mechanisms of the post infection host behavioral change in rats are sexually dimorphic. Here, we report that female rats infected with *Toxoplasma gondii* exhibit greater abundance of messenger RNA for oxytocin and oxytocin receptors in the paraventricular nucleus of the hypothalamus and posterodorsal medial amygdala, respectively. Brain oxytocin is critical for sex-typical social and sexual behaviors in female rodents. The change in oxytocin and its receptor could potentially alter activity in the social salience circuits, leading to a reduction in defensive behaviors and an increase in approach to ambivalent environmental cues. Our results argue that sexually dimorphic neural substrates underpin sexually monomorphic host behavioral change in this host–parasite association.

## Highlights

1. *Toxoplasma* infection enhances mRNA levels for oxytocin in the hypothalamus.
2. *Toxoplasma* infection enhances mRNA for the oxytocin receptor in the medial amygdala.
3. Sexually dimorphic mechanisms underlie behavioral change in two genders.

## 1. Introduction

*Toxoplasma gondii* is a protozoan parasite with a wide host range, including rodents, birds, ruminants, and primates. Rats infected with *Toxoplasma gondii* show a reversal of their innate aversion to cat odors. This behavioral change has been frequently interpreted as a behavioral manipulation of the host by the parasite because *Toxoplasma* requires trophic entry into the cat in to complete its life cycle and also to infect herbivorous hosts that cannot be infected by the asexual carnivorous route. It is postulated that reduced aversion to cat odors can enhance the transmission of the parasite from rats to its definitive felid hosts, although experimental demonstration remains hitherto unattested. Nonetheless, behavioral change in this model system presents a natural perturbation system to understand the neurobiology of fear. Thus, several strands of experimental work have sought to determine proximate mechanisms underpinning the behavioral effects of this parasite [1].

Prior work in male rats shows that *Toxoplasma gondii* infection increases testicular synthesis of testosterone. Testosterone crosses the blood–brain barrier and enhances the transcription of arginine vasopressin in the medial amygdala. These neurons are typically involved in the processing of sexual pheromones in male rats. Their atypical recruitment post infection results in a ‘leakiness’ between circuits involved with sexual behavior and fear. Thus, a fear-inducing semiochemical such as cat urine develops positive valence evoking attraction [1].

Both testosterone and extrahypothalamic arginine vasopressin are sexually dimorphic mediators, with a greater magnitude of expression in males and with specific roles in male-typical behaviors [2,3]. On the other hand, we have recently demonstrated that *Toxoplasma gondii* causes loss of aversion to cat odors in female rats. This change, congruent with males, occurs without corresponding changes in gonadal steroids such testosterone and estradiol. This suggests that the parasitism acts through disparate mechanisms in male and female hosts to achieve the same behavioral change [4]. The nature of these sexually dimorphic mechanisms remains presently unknown.

Oxytocin and arginine vasopressin are closely related nine amino acid peptides with sequence difference of two amino acids [3]. Oxytocin is expressed in female rodents in a sexually dimorphic manner [5]. It is mainly produced by magnocellular neurons in the paraventricular and supraoptic nuclei of the hypothalamus. Axonal transport of oxytocin from these sources leads to the posterior pituitary and eventually outside the brain [6]. In addition, paraventricular nucleus also initiates dendritic release of oxytocin [7] that acts through oxytocin receptors within the brain, including within the medial amygdala [8]. The oxytocin system in the female brain encapsulates nonapeptide signaling through the medial amygdala, akin to arginine vasopressin signaling in the male brain. In this backdrop, we investigate whether *Toxoplasma gondii* infection leads to changes in the oxytocin system in the female brain in this report.

## 2. Results

### 2.1. Oxytocin (OT) mRNA Abundance Postinfection

Oxytocin mRNA abundance in control (*n* = 9) animals and infected (*n* = 9) animals was analyzed in the paraventricular nucleus of the hypothalamus (PVN) and posterodorsal medial amygdala (MePD). Analysis of variance indicated significant main effects for infection status (F_(1, 16)_ = 7.208, *p* = 0.016; abundance in infected group > control) and the brain region (F_(1, 16)_ = 344.9, *p* < 0.0001; PVN > MePD). Interaction between brain regions and infection status did not reach statistical significance (F_(1, 16)_ = 1.38, *p* = 0.257).

**Figure 1:**
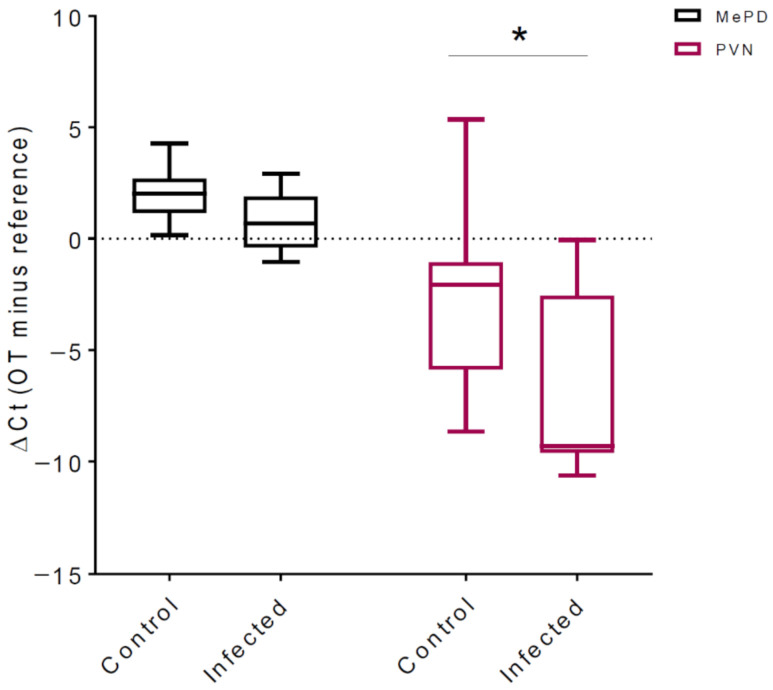
*Toxoplasma gondii* infection increased mRNA abundance of oxytocin (OT) in the posterodorsal medial amygdala (MePD) and the paraventricular nucleus of the hypothalamus (PVN). mRNA isolated from the PVN of infected animals showed a significant increase (decreased ΔCt values) in OT transcription compared with mRNA from the PVN of control animals (* *p* < 0.05). There was no difference in OT levels in the MePD of control and infected animals.

Planned comparison with the Sidak correction demonstrated that *Toxoplasma gondii* infection significantly enhanced oxytocin mRNA abundance in the PVN (Figure 1; t_32_ = 2.639, adjusted *p* = 0.025). Seven out of nine infected animals showed a lower ∆Ct value than the median ∆Ct of the control group, indicating a large effect size (Cohen’s *d* = 0.92). This is also reflected in the higher relative expression ratio for PVN oxytocin post infection (∆∆Ct of means = 3.73; primer efficiency corrected the expression ratio obtained during 10,000 randomizations = 20.8). The infection did not significantly alter oxytocin mRNA abundance in the MePD (t_32_ = 0.853, adjusted *p* = 0.640). The infection-induced increase in oxytocin in the PVN was not due to a generalized facilitation, as indicated by the lack of infection effects on mRNA abundance in the supraoptic nucleus of the hypothalamus (t_16_ = 0.402, *p* = 0.693; data not shown).

### 2.2. Oxytocin Receptor (OTR) mRNA Abundance Postinfection

Oxytocin receptor mRNA abundance in control (*n* = 9) animals and infected (*n* = 8) animals was analyzed in the PVN and MePD. Analysis of variance indicated significant interaction of the infection status with brain regions (F_(1, 15)_ = 7.67, *p* = 0.014). The main effects of infection status (F_(1, 15)_ = 1.67, *p* = 0.216) and brain regions (F_(1, 15)_ = 2.35, *p* = 0.146) did not reach statistical significance.

**Figure 2:**
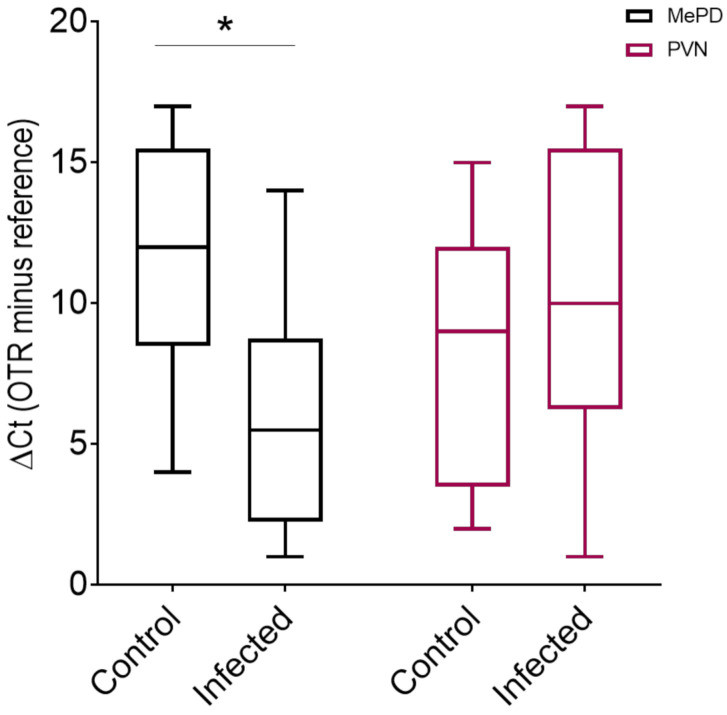
*Toxoplasma gondii* infection increased mRNA abundance of the oxytocin receptor (OTR) in the posterodorsal division of the medial amygdala (MePD) but not in the paraventricular nucleus (PVN) of the hypothalamus. mRNA isolated from the MePD of infected animals showed a significant increase (decreased ΔCt values) in oxytocin receptor (OTR) transcription compared with mRNA from the MePD of control animals (* *p* < 0.05). There was no difference in OTR levels in the PVN of control and infected animals.

A planned comparison with the Sidak correction demonstrated that *Toxoplasma gondii* infection significantly enhanced oxytocin receptor mRNA abundance in the MePD (Figure 2; t_30_ = 2.791, adjusted *p* = 0.018). Seven out of eight infected animals showed a lower ∆Ct value than the median ∆Ct of the control group, indicating a large effect size (Cohen’s *d* = 1.43). This is congruent with the higher relative expression ratio for the MePD oxytocin receptor post infection (∆∆Ct of means = 2.26; primer efficiency corrected the expression ratio obtained during 10,000 randomizations = 3.27). The infection did not significantly alter oxytocin receptor mRNA abundance in the PVN (t_30_ = 0.843, adjusted *p* = 0.647). The infection-induced increase in the oxytocin receptor in the MePD was not due to a generalized facilitation, as indicated by the lack of infection effects on mRNA abundance in the medial preoptic nucleus (t_15_ = 0.476, *p* = 0.641).

## 3. Discussion

We demonstrate that *Toxoplasma gondii* infection of female rats causes an increase in the levels of oxytocin in the paraventricular nucleus of the hypothalamus (PVN). This increase in ligand occurs congruent with increased expression of its receptor in the posterodorsal medial amygdala (MePD). Oxytocin produced within the PVN is released from dendrites and leads to synaptic signaling in distant brain sites, including the medial amygdala, through volume transmission. Thus, the observations reported here suggest that *Toxoplasma gondii* infection enhances oxytocin signaling within the medial amygdala.

Central oxytocin in female rodents is important for sexually dimorphic aspects of sociosexual behaviors. Disruption of oxytocin signaling within the brain causes loss of sexual approach in female mice towards conspecific males [9]. Similarly, oxytocin is important for maternal behavior [10] and is required to form social memories [11]. Pharmacological studies suggest that the medial amygdala is critical for the role of oxytocin in social behaviors [12,13]. The role of the medial amygdala likely involves the binding of oxytocin receptors by the oxytocin produced within the PVN because of sparse extrahypothalamic sources of this nonapeptide and lack of dendritic release from the supraoptic nucleus. Our results suggest that *Toxoplasma gondii* infection causes a potentiation of oxytocin signaling from the PVN to the MePD. This change could potentially alter the activity in the social salience circuits by moving the behavioral output from avoidance to the approach-like behaviors.

Recently, Burg and Hegoburu [14] discussed the role of OT and the OTR in fear response and showed that OT-producing cells are found primarily in the hypothalamus, and these cells project to the central amygdala and secrete oxytocin, which binds to the OTR to determine response to fear stimuli. While their review focused primarily on the central amygdala, the underlying biology of OT secretion seems applicable in our model of innate fear response to predator odor. Thus, their observation that OT-mediated fear response is dependent on the emotional state of the animal provides a new angle to be considered as we endeavor to build a comprehensive understanding of the underlying mechanisms of behavior change post-*Toxoplasma* infection. Additionally, fear extinction in both humans and rodents requires complex interactions between several brain regions and the OT–OTR system. This suggests that while the data presented in this paper focus on OT and OTR expression in the medial amygdala and the hypothalamus, other brain regions that are not primarily involved in fear behavior, including the hippocampus and medial prefrontal complex, may also play a role in the loss of aversion to bobcat odor observed after chronic *Toxoplasma* infection, and warrant investigation to determine whether they play a role in *Toxoplasma*-mediated behavior change [15].

Work on infected male rats supports the possibility of a post infection drift in the approach–avoidance continuum. Nonapeptide arginine vasopressin acting within the MePD reduces avoidance in male rats by recruiting neurons that are typically responsive to female odors. We suggest a similar role of related nonapeptide oxytocin and MePD in female rats. Thus, *Toxoplasma gondii* infection might change host behavior in a sexually monomorphic manner through sexually dimorphic but analogous mechanisms. These results also suggest that innate aversion to predators exists in a dynamic and plastic equilibrium with social behaviors.

## 4. Materials and Methods

### 4.1. Animals and Infection

Female rats of the Wistar Han strain were obtained from InVivos Singapore (7–8 weeks old). Animals were infected with a type 2 strain of *Toxoplasma gondii* (Prugniaud; 5 million tachyzoites suspended in buffered saline, *i.p.*). Corresponding control animals were injected with sterile buffered saline only. Routine management of animals and parasites was similar to that of earlier studies. All mice used in these experiments showed the expected behavioral changes post-chronic *Toxoplasma gondii* infection, and this has been previously published [4]. All experimental procedures were reviewed and approved by the local institutional animal use and care committee.

### 4.2. Quantitative PCR

Animals were sacrificed by rapid decapitation 10 weeks post infection, a time period consistent with the chronic phase of the *Toxoplasma gondii* infection, and after behavioral analysis of aversion to cat odor had been performed. Brain tissue was harvested, flash-frozen in liquid nitrogen, and stored at −80 °C till further analysis. Tissue was sectioned in the coronal plane at 100 µm thickness in a cryotome. Tissue encompassing the paraventricular nucleus of the hypothalamus (bregma −1.80 to −1.88 mm), the supraoptic nucleus of the hypothalamus (bregma −1.80 mm), and the posterodorsal medial amygdala (bregma −3.14 to −3.30 mm) was micro-dissected from slide-mounted brain sections (Appendix A [16]). mRNA was isolated using the standard TRIzol-based method, and reverse-transcribed to cDNA using standard methods (RevertAid First Strand cDNA Synthesis Kit from Fermentas).

cDNA abundance for oxytocin and the oxytocin receptor was quantified using quantitative PCR. A standard method based on SYBR Green dye was used as described before [17]. The PCR cycles needed to reach a predetermined fluorescence threshold at the early linear phase of the amplification were determined (Ct, threshold cycle number). The Ct values for oxytocin (OT) and the oxytocin receptor (OTR) were normalized by subtracting the corresponding geometric mean of the Ct values for three reference genes (HPRT, β-actin, and 18 sRNA). Three technical replicates were used for each determination. The median coefficient of the variation between technical replicates was ≤15%.

The primer sets used for the experiments are indicated below (5′–3′), along with the primer efficiencies estimated using a series of sequentially diluted pooled samples. Oxytocin: TCTGCTGTAGCCCGGATGG and GGAATGAAGGAAGCGCCCTA (efficiency = 94.5%); oxytocin receptor: AAATCCGCACGGTGAAGATGA and CATGTAGATCCACGGGTTGCAG (efficiency = 92.8%); HPRT: AGGCCAGACTTTGTTGGATT and GCTTTTCCACTTTCGCTGAT (efficiency = 96.5%); 18s RNA: ACGGACCAGAGCGAAAGCAT and TGTCAATCCTGTCCGTGTCC (efficiency = 94.5%); β-actin TGTCACCAACTGGGACGATA and GGGGTGTTGAAGGTCTCAAA (efficiency = 90.9%).

### 4.3. Statistical Analysis

Oxytocin mRNA abundance across the paraventricular nucleus and medial amygdala was determined by analyzing the ∆Ct values using two-way ANOVA. Infection status was used as a between-subject and brain region as a within-subject source of variance. In addition, two orthogonal planned comparisons were conducted between control and infected groups for each brain region. The Sidak correction for multiple comparisons was employed. Oxytocin receptor mRNA abundance was similarly analyzed. In addition, relative expression change between control and infected groups was estimated using an efficiency-calibrated model and permutation tests (10,000 randomizations) [18].

## Figures and Tables

**Figure A1:**
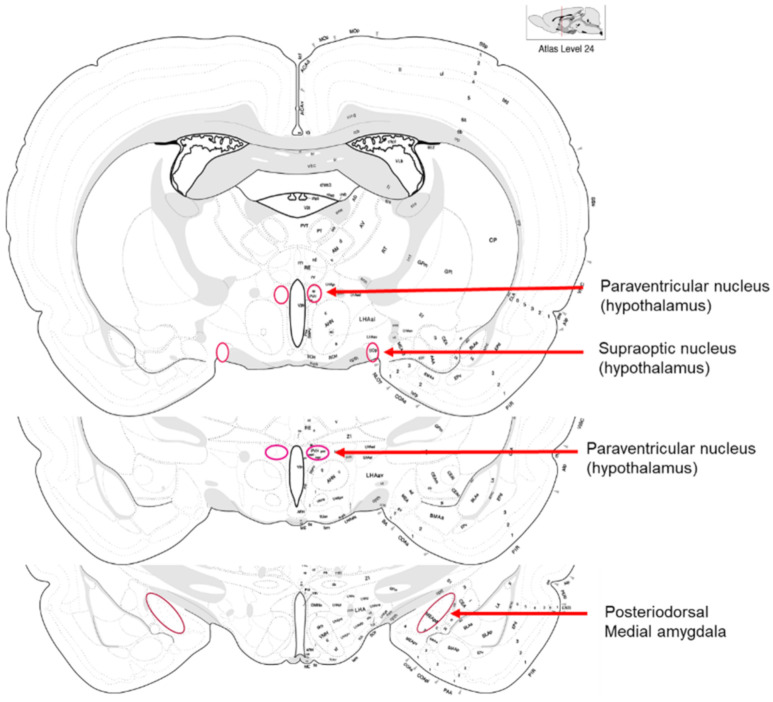
Schematic showing relevant coronal sections and brain regions micro-dissected for quantitative PCR analysis, sections adapted from [16].

## Data Availability

Data are contained within the article.
